# A graphical LASSO analysis of global quality of life, sub scales of the EORTC QLQ-C30 instrument and depression in early breast cancer

**DOI:** 10.1038/s41598-022-06138-2

**Published:** 2022-02-08

**Authors:** Paula Poikonen-Saksela, Eleni Kolokotroni, Leena Vehmanen, Johanna Mattson, Georgios Stamatakos, Riikka Huovinen, Pirkko-Liisa Kellokumpu-Lehtinen, Carl Blomqvist, Tiina Saarto

**Affiliations:** 1 Comprehensive Cancer Center, Helsinki University Hospital and University of Helsinki, Helsinki, Finland; 2 In Silico Oncology and In Silico Medicine Group, Institute of Communication and Computer Systems, School of Electrical and Computer Engineering, National Technical University of Athens, Athens, Greece; 3 Department of Oncology, Turku University Hospital and Faculty of Medicine, University of Turku, Turku, Finland; 4 Faculty of Medicine and Health Technology, Tampere University and Research, Development and Innovation Center, Tampere University Hospital, Tampere, Finland

**Keywords:** Cancer, Oncology

## Abstract

We aimed to (a) investigate the interplay between depression, symptoms and level of functioning, and (b) understand the paths through which they influence health related quality of life (QOL) during the first year of rehabilitation period of early breast cancer. A network analysis method was used. The population consisted of 487 women aged 35–68 years, who had recently completed adjuvant chemotherapy or started endocrine therapy for early breast cancer. At baseline and at the first year from randomization QOL, symptomatology and functioning by the EORTC QLQ-C30 and BR-23 questionnaires, and depression by the Finnish version of Beck's 13-item depression scale, were collected. The multivariate interplay between the related scales was analysed via regularized partial correlation networks (graphical LASSO). The median global quality of life (gQoL) at baseline was 69.9 ± 19.0 (16.7–100) and improved to 74.9 ± 19.0 (0–100) after 1 year. Scales related to mental health (emotional functioning, cognitive functioning, depression, insomnia, body image, future perspective) were clustered together at both time points. Fatigue was mediated through a different route, having the strongest connection with physical functioning and no direct connection with depression. Multiple paths existed connecting symptoms and functioning types with gQoL. Factors with the strongest connections to gQoL included: social functioning, depression and fatigue at baseline; emotional functioning and fatigue at month 12. Overall, the most important nodes were depression, gQoL and fatigue. The graphical LASSO network analysis revealed that scales related to fatigue and emotional health had the strongest associations to the EORTC QLQ-C30 gQoL score. When we plan interventions for patients with impaired QOL it is important to consider both psychological support and interventions that improve fatigue and physical function like exercise.

Trial registration: http://www.clinicaltrials.gov/ (identifier number NCT00639210).

## Introduction

The incidence of early breast cancer is high and the prognosis is in most cases with modern treatments excellent^1^ which highlights the importance of understanding the factors which have effect on health related quality of life (QOL) after breast cancer treatments among survivors. The QOL concept has been highly influenced by WHO:s definition of health as health is a state of complete physical, mental, and social well-being, and not merely the absence of disease and infirmity^2^. The concept of health related quality of life has been proposed to refer to the aspects of quality of life that are related to disease or its symptoms, however, both of these concepts are often used interchangeably^3^.

During the active treatment period, side-effects influence the QOL of the early breast cancer patients and QOL has been found to be impaired as compared to the general population^4,5^. During the first 12 months from the surgery spontaneous recovery will occur as side-effects usually decrease after chemotherapy cessation. Side-effects related to the hormonal changes after chemotherapy and/or start of endocrine therapy also gradually diminish^6–9^. However, in a previous study we showed that the QOL of breast cancer survivors was still impaired compared to a control population even 10 years after primary treatment^10^. It is therefore important to have a better understanding of the factors associated to impaired QOL of the breast cancer survivors during the rehabilitation period to be able to plan correct interventions.

The EORTC QLQ-C30 instrument for assessing health related QOL of cancer patients has been widely used in oncological studies over the last decades^11–24^**.** It consists of nine multi-item scales: five functional scales, three symptom scales, a global health and QOL scale and single item symptom measures^25^. This instrument together with breast cancer specific module BR23^26^ (currently BR45^27^) takes into account the specific side-effect which are related to breast cancer treatments also during the rehabilitation period. As endocrine treatment lasts several years the systemic therapy side-effects are important negative factors to influence QOL^23^. Depression is known to be a common symptom among breast cancer patients^9^ and it needs to be also taken into account when studying reasons for impaired QOL. Depression is not included separately to the EORTC QLQ-C30 instrument.

To be able to target interventions for patients with impaired quality of life knowledge of which factors are the most important would be useful. BReast cancer and EXercise (BREX) trial is a prospective randomized controlled trial where patients with early breast cancer were randomized either to the 12-months exercise intervention group or the control group. We analysed the associations between depression (BDI), global health/quality of life (gQoL) and selected symptom and functioning scales of the QLQ-C30 and BR23 questionnaires during the first year of the rehabilitation period in BREX trial, using a network analysis method based on the network theory of mental disorders. According to Borsboom^28^ this theory offers an alternative conceptualisation to the classical model of “certain illnesses causing certain symptoms”, focusing instead on the causal relations between the symptoms mediated by different biological, psychological and societal mechanisms. Symptoms form networks that can, if sufficiently strong, become self-sustaining and thereby stuck in a disorder state. Mental disorders can thus be understood as alternative stable states of strongly connected symptom networks.

In quality of life research, the observed/reported symptoms typically correlate with each other and it is important to understand their interplay to grasp the causal complexity of the QoL outcome, e.g., in cancer patients. We therefore found the network theory the best conceptual basis for the present work and chose our method of analysis accordingly^29–32^. Network analysis has previously been applied to examine the connections both at item and domain level of the 36-item Short Form Health Survey (SF-36), demonstrating its usefulness in health related QOL research^32^. The analysis revealed that the global structure of this QOL instrument was dominant in all networks supporting the validity of this questionnaire's subscales.

In the current study, the perceived health status as reflected by the gQoL item of the EORTC QLQ instrument is viewed as the outcome of complex interactions among symptoms and functioning measured by this instrument and the depression score measured by the BDI questionnaire.

The aim is to (a) explore the relationships among functions and symptoms, and how they are connected with gQoL, (b) to explore how these relationships change from baseline to one year of follow-up and (c) examine the importance of each scale in the network based on centrality analysis. The analysis was done in 487 breast cancer patients participating in an exercise intervention study during their first year of follow-up.

## Patients and methods

### Patients

The BREX study population consisted of 573 women aged 35–68 years, who had recently completed adjuvant chemotherapy or started endocrine therapy of early breast cancer. Patients were randomized to an exercise intervention group or to the control group as described in detail in previous publications. Randomization took place when patients completed adjuvant chemotherapy or started endocrine therapy of early breast cancer. One of the main endpoints of this trial, the effect of exercise intervention on the QOL after 1 and 5 years from randomization, has been published elsewhere^33,34^.

The 1-year exercise intervention consisted of both supervised and home training. In this study we analyse the whole population not divided based on the intervention, since no significant QOL-difference was found between the two groups^35^.

Detailed inclusion and exclusion criteria and the flow diagram of the participants through five years are presented in previous publications^33–35^. In the present study we have used the follow up data from baseline and the one-year follow-up visit. From the 573 patients initially enrolled, 80 patients discontinued the trial during the first year (as they did not meet the inclusion criteria at baseline, or for personal reasons)^35^ and were excluded from the analysis. Additionally, 6 patients that participated in the study only once during the first year were removed, leaving 487 patients for the final analysis.

In the final dataset, all questionnaires were missing for 10 and 20 patients at baseline and month 12 respectively. The percentage of patients with missing questionnaire scale scores was 4% at both time points.

The study was conducted in accordance with the Declaration of Helsinki. The study protocol was approved by the ethical committee of the Helsinki University Hospital. The patients received oral and written information, and a written informed consent was obtained from all patients. All experiments were performed in accordance with relevant guidelines and regulations.

The BREX trial is registered in the Helsinki and Uusimaa Hospital District Clinical Trials Register (www.hus.fi) (trial number 210590) and at http://www.clinicaltrials.gov/ (identifier number NCT00639210).

### Methods

Clinical investigations*,* including basic laboratory safety tests and radiological examinations, were done according to the usual follow-up practice. The medical history of the patients was surveyed during the baseline visit for the BREX study after adjuvant treatment and included a medical examination and laboratory tests. In addition, the patients filled out a questionnaire covering QOL, basic demographics, and lifestyle issues. The same measurements were repeated after one year.

*QOL and mental well-being:* QOL was measured by the EORTC QLQ-C30^25^ with the breast cancer module supplement (BR-23)^26^. EORTC QLQ-C30 It consists of nine multi-item scales: five functional scales (physical, role, cognitive, emotional, and social); three symptom scales (fatigue, pain, and nausea and vomiting), and a global health and QOL scale. The Global health/QOL scale describes patients’ subjective quality of life and is based on two questions about physical condition and overall QOL and is abbreviated gQoL here. Several single-item symptom measures are also included (e.g., dyspnoea, appetite loss, sleep disturbance, constipation, and diarrhoea)^25^. Breast cancer specific module BR23 was introduced in 1996 to assess the impact of common breast cancer treatment modalities (surgery, chemotherapy, radiotherapy, or endocrine treatment) upon women’s well-being^26^.

Depressive symptoms were assessed by the Finnish modified version of Beck's 13-item depression scale (BDI)^36^.

### Missing data imputation

Missing data were imputed using the *copyMean* method in R package *longitudinalData*. The method is designed for longitudinal data and is a variant of linear interpolation accounting for the shape of the mean population trajectory^37^. This method has demonstrated effectiveness, exceeding or equalling the performance of other single imputation methods (e.g. cross-sectional mean or median, trajectory mean or median, last occurrence carried forward, linear or spline interpolation)^37^.

### Univariate analysis

The correlation between gQoL with the other QLQ-C30 scales, QLQ-BR23 scales and BDI score were measured with the Spearman correlation coefficient. A correlation (absolute value) from 0.10 to 0.29 was classified as weak, from 0.3 to 0.49 as moderate, and ≥ 0.50 as strong. Adjusted *p*-values were obtained using Benjamini and Hochberg method. A *p*-value < 0.05 was considered statistically significant^38,39^.

### Network construction

Symptoms and functions related to quality of life are typically associated with each other. However, an observed association between two variables might be misleading, i.e. not reflecting a genuine interaction between them. Such a spurious association can arise from a third variable (typically called confounder or controlling variable*)* that is a common cause of the two variables^29^ The aims of the present work was to remove spurious associations and reveal the structure of the genuine interactions among symptoms and functions. To achieve this, we conducted an analysis of partial correlations visualized as networks (also known as Gaussian Graphical models (GGM)) for baseline and 12-month follow up. Each network consists of separate entities, termed ‘nodes’, and connections between them, termed ‘edges’. In our analysis, ‘nodes’ represent the BDI score, the gQoL and QLQ-C30 and BR23 symptom and functioning scales selected based on the magnitude of the Spearman correlation with gQoL (average rho between baseline and one year follow-up > 0.3). An ‘edge’ reflects the association between two nodes after controlling for all other nodes in the network (partial correlation). Edge thickness is proportional to the magnitude of the partial correlation. Network analysis helps to tackle complex associations among a large number of entities and enables the visual and quantitative perception of such associations^30^. Compared to association networks, where each edge represents a zero-order correlation, partial correlation networks discern predictive effects and takes us one step closer to causal relationships. More precisely, in a partial correlation network, the edges connected to a single node are analogous to the regression coefficients obtained in a multiple regression model, where the dependent variable is the node under consideration^30^ Moreover, representing partial correlations, the edges are considered indicative of causal relations between the connecting nodes^30,40^.

We followed standard procedures as described in^29,41^ using the R package *qgraph*^41^. Following suggestions in Johnson and Creech (1983), Norman (2010), Sullivan and Artino (2013), Zumbo and Zimmerman (1993)^42–45^, we treated scales with less than 7 levels as ordinal, otherwise as having a continuous interval scale. A correlation matrix was initially computed using the *cor_auto* function, consisting of Pearson correlations between continuous scales, polyserial correlations between continuous and ordinal scales, and polychoric correlations between ordinal scales. The use of polychoric correlations in the case of ordinal data has proved efficient when estimating partial correlation networks^46^. Following, we employed the graphical LASSO (*EBICglasso* algorithm)^47^ to perform regularized estimation of the partial correlation networks^47^. Regularization serves two purposes. The first is to compute partial correlations (from zero-order correlations) between all pairs of variables, i.e. the association between two variables that remains after eliminating the influence of all other variables. The second is to shrink small, likely spurious associations to zero, returning a sparse network, i.e. a network that is not fully connected, but contains as few connections as possible to parsimoniously explain the observed covariance among variables^30,41^. It is noted that LASSO will always return a sparse network, regardless of the density of the underlying true network^31^.We find the assumption of a sparse underlying true network plausible in the case of a quality of life network. We expect each symptom to directly interact with certain symptoms and functions and we don’t consider likely everything to interact with one another. The level of sparsity of the estimated networks was selected by minimizing the extended Bayesian information criterion (EBIC). The EBIC tuning hyperparameter, γ, was set to the recommended value 0.5^48^, that results in a sparser and, hence, more interpretable network and avoids most spurious edges. The method does not require significance testing. Networks were visualized according to Fruchterman-Reingold layout^49^. Nodes that are connected with more and stronger edges tend to be shown near each other^50^. The approach allows a visual inspection of the grouping of nodes^51,52^.

### Modularity analysis

We investigated the existence of subgroups of nodes (communities) using the R package igraph^53^. A community is defined as a subgroup of nodes within a network characterized by denser connections between its members than with the rest of the network^54^. We used three algorithms to identify communities, namely *optimal*^55^, *spinglass*^56,57^ and *walktrap*^58^. The community structure with the higher modularity score was selected. Modularity is a widely used metric that expresses the quality of a network’s division into communities^59^. It depends only on the choice of the communities (or clusters) in the network. The higher the modularity score of a community structure, the denser the within-community connectivity compared to between-community connectivity, and, hence, the better the quality of the structure. In a weighted network the modularity of a specific division of the network into communities, is the sum over all communities of the total weight of the edges in a community minus the total weight expected by chance in the community^53,60^. A modularity score approximately 0.3 or more indicates a good community structure^60^ When the modularity score is zero, the within–community connectivity does not differ from the one expected by chance^60,61^. The maximum value of modularity is 1.

### Node importance

Node importance was evaluated with the centrality indices of strength, closeness and betweenness^62–65^. *Strength* sums the weights of the edges (absolute values) that stem from the node. A node with high strength has a strong direct connectivity with other nodes. *Closeness* is the inverse of the sum of the distances of the node of interest from all the other nodes in the network. The distance between two nodes is the length of the shortest path between them, i.e., the sum of the inverse of the absolute edge weights that comprise the path. A node with high closeness will affect other nodes quickly or will be quickly affected by changes in another node, via both direct and indirect connections. *Betweenness* is defined as the number of geodesics (between all pairs of nodes) that pass through the node of interest. A node with high betweenness is important in connecting other nodes.

### Network stability and accuracy

We assessed the accuracy of networks’ structure using the R package *bootnet*, following the guidelines^41^. We performed three types of analysis. Firstly, we assessed network stability by reconstructing the network in case-dropping bootstraps of decreasing size. If a large proportion of cases is allowed to be dropped, before the order of edge weights or centralities becomes unstable, the results are considered interpretable. We computed the correlation stability coefficient (CS-coefficient) that expresses the maximum proportion of dropped cases, such that the correlation between the original and the bootstrap network is at least (cor =) 0.7 with a 95% probability. It is suggested *CS*-coefficient to be at least 0*.*25 and preferably above 0*.*5 for edge weights or centralities to be considered stable^41^. Secondly, we investigated the sampling variability in edge-weights by estimating 95% confidence intervals (CIs) around them via non-parametric bootstrap (resampling with replacement). Thirdly, we tested for significant differences between different edge weights and different node strength-centralities via non-parametric bootstrap. The latter method does not include correction for multiple testing. We computed 1000 bootstrapped networks for each analysis.

### Temporal network comparison

We compared the networks at baseline and month 12 using two types of analysis. We first computed the Spearman correlation between the edge weights of the two networks. Following this we evaluated the differences between the networks by using the permutation-based statistical test in R package *NetworkComparisonTest*^66^. By performing random permutations, the method produces a reference distribution of differences under the null hypothesis that the networks are equal. The observed difference is evaluated against the reference distribution yielding the *p*-value. The following were tested for significant differences between the two time points: edge weights, node strength-centralities, global strength (absolute sum of edge weights) and network structure (maximum absolute difference in edge weights)^66^. Regarding edge weights and node strength-centralities, the test was performed without correction for multiple testing. Because the networks refer to one patient group, we applied the dependent version of the test. It is noted that the test has not been validated for dependent data. We computed 5000 permutations. A significance level of 0.005 was considered. Finally, we calculated the difference in edge weights between the two time points by subtracting the corresponding adjacency matrices (absolute values). The differences were visualized as a network using the R package *qgraph*.

### Ethical approval and informed consent

This study involves human participants and the experiments comply with the current laws of the countries in which they were performed. The patients received oral and written information, and a written informed consent was obtained from all patients.

## Results

### Patient characteristics

The median age of patients was 52 years (range 35–68). One hundred and eighty five (35%) patients were lymph node positive. Two hundred thirty-five (48%) patients were operated with breast conserving surgery and two hundred fifty-two (52%) with mastectomy. Four hundred forty five (91%) patients received chemotherapy and four hundred and five (83%) received endocrine therapy. Two hundred and sixty-two patients were postmenopausal and two hundred and twenty-five (46%) were premenopausal. Baseline characteristics of the 487 analysed patients are described in the Supplement Table 1. Table 1 presents QOL and depression at baseline and 12-month follow up in the whole population and separately according to the randomization groups.

**Table 1:** Health related quality of life at baseline and 12-month follow up measured by the EORTC QLQ-C30 and -BR23 and the BDI questionnaires.

	Mean ± SD (range)	
	Baseline	Month 12
*EORTC-QLQ-C30 scales*		
Global QoL	69.9 ± 19.0 (16.7–100)	74.9 ± 19.0 (0–100)
Physical functioning	82.4 ± 15.7 (33.3–100)	85.0 ± 15.0 (20–100)
Role functioning	86.7 ± 19.2 (0–100)	89.8 ± 18.0 (0–100)
Social functioning	87.4 ± 19.4 (0–100)	93.9 ± 15.1 (0–100)
Emotional functioning	82.4 ± 17.5 (0–100)	83.9 ± 17.6 (0–100)
Cognitive functioning	84.6 ± 19.5 (0–100)	85.1 ± 19.2 (0–100)
Fatigue	72.6 ± 18.6 (0–100)	77.3 ± 18.9 (0–100)
Insomnia	69.3 ± 29.2 (0–100)	73.7 ± 30.0 (0–100)
Pain	83.0 ± 18.9 (16.67–100)	81.6 ± 22.0 (0–100)
Nausea and vomiting	96.8 ± 9.6 (16.67–100)	98.0 ± 6.7 (33.33–100)
Dyspnea	93.6 ± 14.7 (0–100)	94.0 ± 15.8 (0–100)
Appetite loss	95.0 ± 14.7 (0–100)	97.3 ± 10.1 (33.33–100)
Constipation	88.9 ± 19.1 (0–100)	89.1 ± 19.7 (0–100)
Diarrhea	94.4 ± 14.7 (0–100)	94.7 ± 14.4 (0–100)
Financial difficulties	88.7 ± 21.9 (0–100)	94.2 ± 17.4 (0–100)
*EORTC-QLQ-BR23 scales*		
Body Image	63.7 ± 27.4 (0–100)	74.9 ± 25.7 (0–100)
Sexual functioning	29.7 ± 26.2 (0–100)	34.1 ± 27.5 (0–100)
Sexual enjoyment	39.5 ± 36.2 (0–100)	43.7 ± 36.2 (0–100)
Future perspective	55.0 ± 28.8 (0–100)	64.2 ± 26.6 (0–100)
Systemic therapy side effects	78.4 ± 13.8 (27.78–100)	83.5 ± 12.0 (16.67–100)
Breast symptoms	81.4 ± 16.3 (16.67–100)	88.2 ± 14.2 (16.67–100)
Arm symptoms	82.0 ± 18.2 (11.11–100)	81.8 ± 18.7 (11.11–100)
Upset by hair loss	59.3 ± 35.5 (0–100)	92.5 ± 22.3 (0–100)
*BDI*		
Depression	35.4 ± 3.8 (11–39)	35.9 ± 3.9 (14–39)

*EORTC-QLQ-C30*: Quality of Life Questionnaire-Core instrument (QLQ-C30) of the European Organization for Research and Treatment of Cancer (EORTC) study group on quality of life of cancer patients, *EORTC-QLQ-BR23*: BReast cancer specific module of the EORTC*-*QLQ, *BDI*: Finnish modified version of Beck's 13-item depression scale, *QOL:* Quality Of Life, *SD*: standard deviation.

The depression score and the symptom scores have been reversed to follow the functioning scales interpretation, i.e. a higher score indicates a lower level of symptoms and a better state of the patient.

### Univariate analyses

Correlations of gQoL with symptoms, functioning and depression at baseline and month 12 are reported in Table 2. All scales showed statistically significant correlations with gQOL, with the exception of symptom scales ´diarrhea´ and ´upset by hair loss´ at baseline.

**Table 2:** Correlations (Spearman’s rho) of global health/QOL with symptoms, functioning and depression at baseline and 12-month follow up.

	Global health/QOL	
	Baseline	Month 12
*EORTC-QLQ-C30 scales*		
Physical functioning	0.48***	0.53***
Role functioning	0.50***	0.52***
Social functioning	0.45***	0.45***
Emotional functioning	0.45***	0.52***
Cognitive functioning	0.38***	0.46***
Fatigue	0.55***	0.58***
Insomnia	0.33***	0.33***
Pain	0.34***	0.54***
Nausea and vomiting	0.26***	0.30***
Dyspnea	0.23***	0.25***
Appetite loss	0.21***	0.21***
Constipation	0.14*	0.16**
Diarrhea	0.07	0.19***
Financial difficulties	0.28***	0.34***
*EORTC-QLQ-BR23 scales*		
Body image	0.43***	0.42***
Sexual functioning	0.21***	0.31***
Sexual enjoyment	0.25***	0.33***
Future perspective	0.34***	0.48***
Systemic therapy side effects	0.35***	0.47***
Breast symptoms	0.27***	0.26***
Arm symptoms	0.28***	0.39***
Upset by hair loss	0.16	0.27***
*BDI*		
Depression	0.52***	0.56***

*EORTC-QLQ-C30*: Quality of Life Questionnaire-Core instrument (QLQ-C30) of the European Organization for Research and Treatment of Cancer (EORTC) study group on quality of life of cancer patients, *EORTC-QLQ-BR23*: BReast cancer specific module of the EORTC*-*QLQ, *BDI*: Finnish modified version of Beck's 13-item depression scale, *QOL:* Quality Of Life.

The depression score and the symptom scores have been reversed to follow the functioning scales interpretation, i.e. a higher score indicates a lower level of symptoms and a better state of the patient.

*P*-values for correlation coefficients were calculated by two-sided t-test and were adjusted using Benjamini and Hochberg method.

*p < 0.05; **p < 0.01; ***p < 0.001.

At baseline, gQoL correlated strongly (> 0.5) with fatigue, depression and role functioning. Moderate correlations (0.3–0.5) were observed between gQoL with physical, emotional, social and cognitive functioning, body image, future perspective, pain and systemic therapy side effects. At month 12, gQoL correlated strongly with fatigue, depression, physical, emotional and role functioning, as well as pain. Moderate correlations were observed between gQoL with future perspective, systemic therapy side effects, social and cognitive functioning, body image, arm symptoms, financial difficulties, insomnia, sexual functioning and enjoyment and nausea/vomiting.

Only scales characterized by medium to strong correlations with gQoL (average rho over first year > 0.3) are considered in the subsequent network analysis. These are fatigue, depression, all the functional scales (emotional functioning, physical functioning, role functioning, social functioning, cognitive functioning), pain, insomnia, financial difficulties, body image, future perspective, systemic therapy side-effects, and arm symptoms.

### Network structure

**Figure 1:**
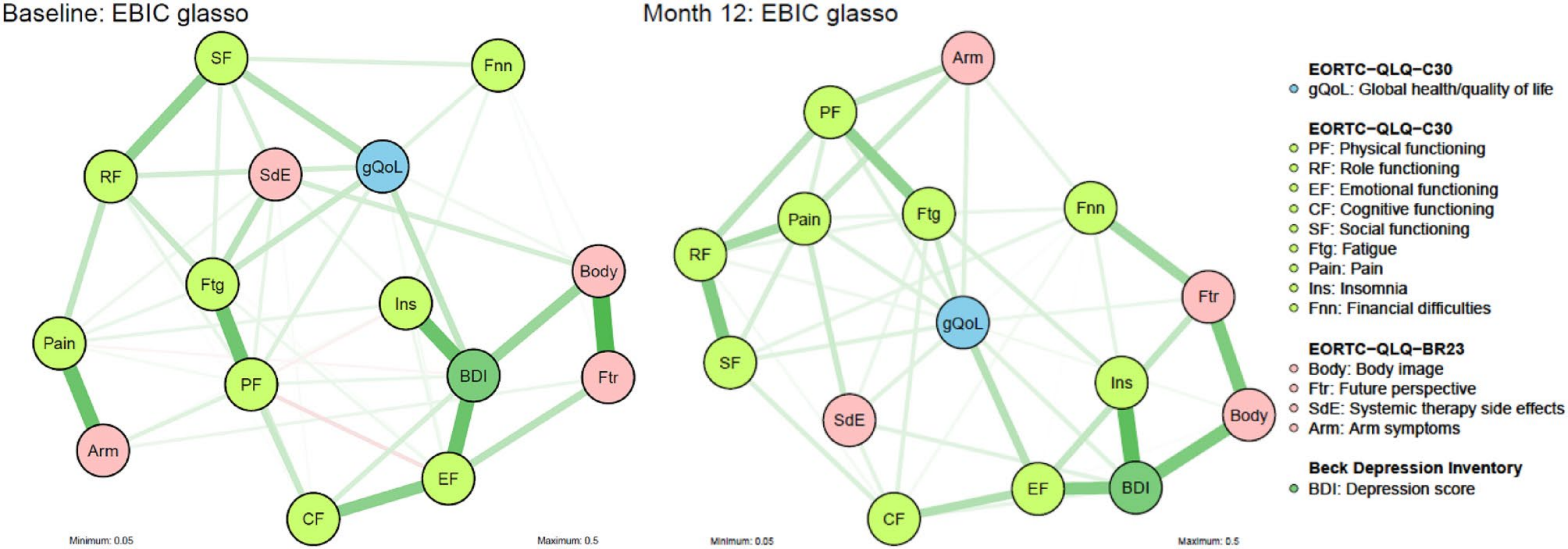
Networks constructed via graphical LASSO visualizing the regularized partial correlations between global health/QoL, symptoms, functioning and depression, measured by EORTC-QLQ C30, EORTC-QLQ B23 and BDI questionnaires, at baseline and 12-month follow up. Green edges represent positive partial correlations and red edges negative ones. Thicker and more saturated edges represent stronger partial correlations. Edges with absolute weight above 0.05 are displayed. The distance between two nodes reflects the absolute edge weight between them (Fruchterman–Reingold layout). All edge weights are reported in Supplement Table 3. The depression score and the symptom scores have been reversed to follow the functioning scales interpretation, i.e. higher score indicates a lower level of symptoms and a better state of the patient.

Figure 1 depicts the networks between gQoL, functioning, symptoms and depression, at baseline and 12-month follow up. Relatively dense regularized networks were constructed by EBIC glasso, having 74 (70.5%) and 68 (64.8%) nonzero edges, out of 105 possible ones, at baseline and month 12, respectively. Analysis of network stability and accuracy (Supplement Table 2 and Supplement Figs. 1–3) indicate that the networks are fairly stable and accurately estimated and support the following findings.

Edge weights (absolute values) range between 0.002 and 0.358 at baseline and between 0.002 and 0.333 at month 12. The strongest associations that are consistently evident in both networks are between insomnia—depression (edge weights: M0:0.307, M12:0.334), emotional functioning—depression (M0:0.310, M12:0.264), body image—depression (M0:0.225, M12:0.276), future perspective—body image (M0:0.358, M12:0.277), physical functioning—fatigue (M0:0.301, M12:0.241) and role functioning—social functioning (M0:0.240, M12:0.271). Almost all edges that disappear in month 12 have low weights (< 0.09) at baseline. A characteristic exception is between physical functioning—cognitive functioning (M0:0.143, M12:0). All edge weights are reported in Supplement Table 3.

At baseline, gQoL is connected directly by edges of very low weight (< 0.03), or not connected at all, with the symptom scales systemic therapy side-effects, pain, arm symptoms, and insomnia. Characteristic paths that connect systemic therapy side-effects with gQoL go through fatigue and social functioning, the main path that connects pain and arm symptoms with gQoL goes through role functioning, whereas insomnia is connected with gQoL primarily through depression. Furthermore, no direct connection is evident between gQoL and cognitive functioning or future perspective. At month 12 the partial correlation of future perspective and the symptom scales of systemic therapy side-effects, pain, arm symptoms with gQoL seems to increase.

Based on visual inspection, scales related to mental health (depression, body image, future perspective, emotional functioning, cognitive functioning, insomnia) are clustered together at both time points. The modularity analysis identified four sub-groups at baseline and two sub-groups at month 12. However, the modularity scores, Q, are low (M0: Q = 0.22, M12: Q = 0.23) indicating that the networks do not have a strong community structure. At baseline the four detected sub-groups are: (1) arm symptoms—pain, (2) future perspective—body image—financial difficulties (3) insomnia—depression—emotional functioning—cognitive functioning and (4) social functioning—role functioning—physical functioning—systemic therapy side effects—fatigue—gQoL. At month 12 the above subgroups are merged into two: (1) Future perspective—body image—financial difficulties—insomnia—depression—emotional functioning—cognitive functioning and (2) arm symptoms—pain—social functioning—role functioning—physical functioning—systemic therapy side effects—fatigue—gQoL.

### Node importance

Stability analysis (Supplement Table 2) indicates that the order of node strength is stable under sub setting cases (*CS*(cor = 0.7) > 0.5), whereas the stability of closeness is moderate (*CS*(cor = 0.7) > 0.25), for both time points. Contrarily, CS-coefficients for betweenness fail to reach the minimum threshold of 0.25. Thus, results are interpretable for node strength and closeness.

**Figure 2:**
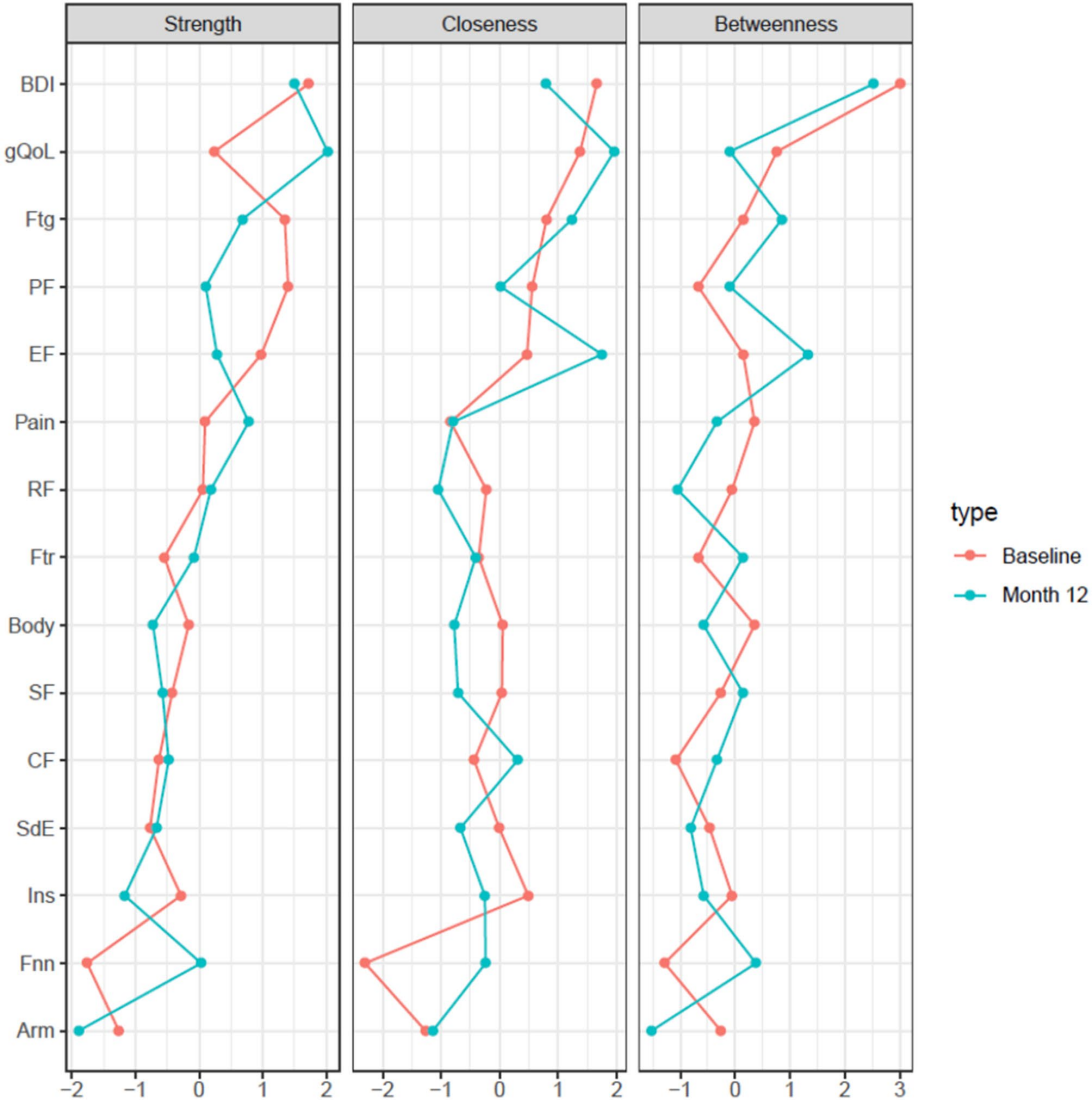
Centrality plots for graphical LASSO networks at baseline and 12 month follow up. The strength, closeness and betweenness of each node are depicted as standardized z-scores. Abbreviations: gQoL: Global Quality of Life, PF: Physical functioning, RF: Role functioning, SF: Social functioning, CF: Cognitive functioning, EF: Emotional functioning, BDI: Depression score, Ftg: Fatigue, Ftr: Future perspective, Body: Body image, Pain: Pain, SdE: Systemic therapy side effects, Ins: Insomnia, Arm: Arm symptoms, Fnn: Financial Difficulties.

Figure 2 shows the centrality indices (standardized *z*-scores.) at baseline and month 12. At baseline, the most central node is depression, as it has the highest strength and closeness. Important nodes in terms of strength are also physical functioning, fatigue and emotional functioning and in terms of closeness are gQoL, fatigue, physical functioning, insomnia, and emotional functioning. The least important nodes are arm symptoms and financial difficulties. At 12 months follow up, the most important nodes in terms of strength are gQoL, BDI, fatigue and pain and the least important nodes are arm symptoms and insomnia. In terms of closeness the most important nodes are gQoL, emotional functioning, fatigue and depression and the least important is arm symptoms.

### Temporal network comparison

**Figure 3:**
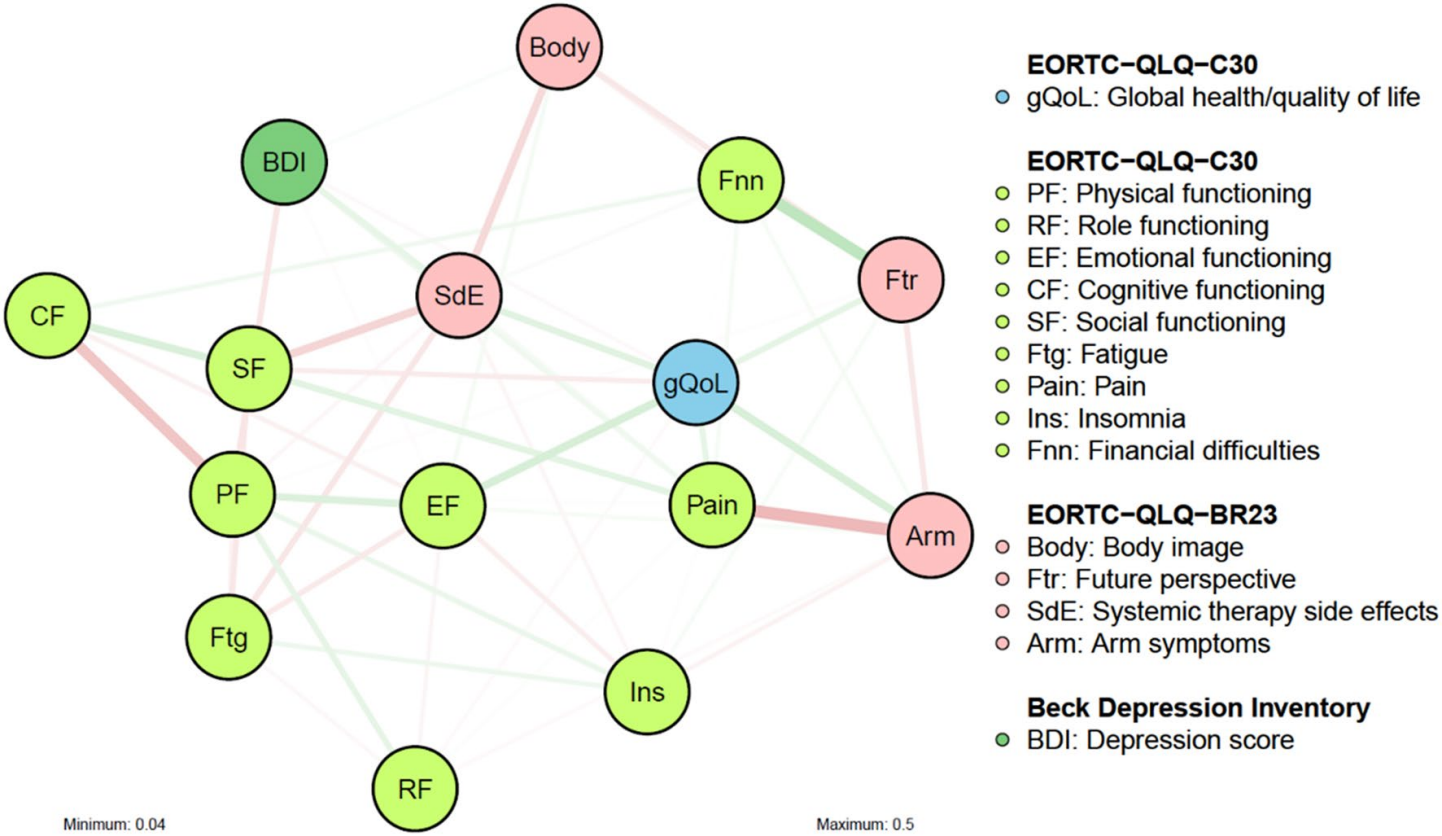
Difference network. Each edge corresponds to the difference in the absolute value of edge weights between the baseline and month 12. Green lines represent an increase in the absolute value of edge weight from baseline to month 12 and red lines a decrease. Thicker and more saturated lines represent a higher difference. Differences of absolute value above 0.04 are displayed.

The Spearman correlation between the edge weights of the networks at baseline and month 12 is rho = 0.62 (*p* < 0.001), indicating a moderate similarity of the two networks. Figure 3 highlights the differences in edge weights between the two networks. The difference network is characterized by weak connections, with the strongest one being 0.17 (absolute value/ referring to edge pain-arm symptoms). The mean difference in edge weight is 0.04. According to the network comparison test, no edge differed significantly at the 0.005 level between baseline and Month 12. In terms of strength centrality, a statistically significant increase was observed only for financial impact. The *p*-values are given in Supplement Tables 4 and 5. Finally, no statistically significant difference was found for network structure (*p* = 0.30) and global strength (*p* = 0.16). This means that the network is fairly stable over time. It is noted, however, that we cannot exclude the possibility that the test does not have enough statistical power to detect significant changes in our data.

## Discussion

Quality of life describes a person’s ability to enjoy normal life activities. The baseline in this study reflects the QOL of breast cancer survivors who have recently completed chemotherapy or started endocrine therapy. The follow-up time in the present analysis is one year from randomization. We know from the literature that the QOL of breast cancer survivors decreases during and after adjuvant treatments^5–8^ but will usually improve during the first year, as published also from this patient cohort earlier^34^.

To our knowledge this is the first study of the interplay and correlation structure between BDI depression scale and the EORTC QLQ-C30 and BR23 functional, symptom and gQoL scales in breast cancer patients using network analysis, namely graphical LASSO method. In addition to studying the association network between the different items in the EORTC QLQ-C30-scale we also included the BDI-questionnaire, since depression is common during the early rehabilitation after a cancer diagnosis^67^.

The present analysis treats QOL as a network of interacting components according to the network theory described in the Introduction, using psychometric scales/domains as components. The network visualizes (regularized) partial correlations between pairs of psychosocial scales, with the effect of the rest of the scales removed. Even though cross-sectional analysis of this type (weighted but undirected networks) cannot confirm causality among scales, the presence of a connection between two components of the network suggests a plausible candidate for a relationship between cause and effect. On the other hand, the absence of a connection implies that one component cannot have caused the other.

Scales to be included in the network analysis were selected based on Spearman test. Most of the functional scales showed medium to strong correlation with gQoL, but as trial inclusion took place after chemotherapy certain side-effects that were especially related to surgery (breast symptoms) or chemotherapy (nausea, hair loss, diarrhea) showed weak or even insignificant correlation with gQoL and did not exceed the threshold for inclusion in the network. Some side-effects had a stronger correlation to the gQoL after one year as presented in Table 2. One explanation may be that long-term side-effects are more difficult to tolerate than short-term reversible symptoms.

Depression and fatigue are known to be strong predictors for breast cancer patients’ QOL^34,67^ as published also from this study at baseline in 2011^34^. This was also the case after one year as we report here. Based on network analysis the connection of depression with gQoL at baseline is strong and direct, while after 1 year two major paths are evident, a direct one and a path through emotional functioning. However, since the temporal differences in network did not reach statistical significance one should interpret this apparent difference with caution. At baseline also social functioning had a strong correlation with gQoL. Fatigue (measured with EORTC QLQ-C30) had a strong and direct correlation with gQoL both at baseline and after one year from randomization. Fatigue is defined as a distressing, persistent, subjective sense of physical, emotional, and/or cognitive tiredness related to cancer or cancer therapy, that is not proportional to recent activity and interferes with usual functioning^68^. Fatigue has a multidimensional nature. In many studies fatigue has been associated to mental health^69–71^ and psychological interventions have been suggested. In the present study, however, fatigue was strongly associated to physical function both at baseline and after 12 months. In a systematic review Abrahams et al. also reported that fatigue has a significant correlation with physical functioning in 10 out of 12 studies^69^. Based on visual inspection and the modularity analysis, scales related to mental health are clustered together at both time points while fatigue is associated with gQoL through other links. Our study supports the importance of physical function for fatigue and suggests that fatigue may be a target for intervention as several meta-analyses have shown that it can be eased with exercise^72–76^. In a previous publication we showed that both fatigue and physical function scores of the participants in the present study improved during the first year simultaneously with improving exercise habits and measurements of cardiorespiratory fitness^35^.

Changes in nodes of high centrality are expected to have considerable influence over the network. The high centrality of depression and fatigue in the networks suggests they would be important targets for intervention to improve QOL of breast cancer patients during the treatment and the early follow-up period. However, centrality indices in cross-sectional, undirected networks can only generate hypothesis^77^. A node may have high centrality because it influences many other nodes or it is influenced by many other nodes in the network. Interventions targeting highly central nodes can be effective only if these nodes causally influence other nodes in the network, i.e. cause other symptoms or impair functioning, and not vice versa.

The strengths of the present study include a large sample of patients and the fairly homogenous treatment including endocrine and cytotoxic adjuvant therapies in most of the cases. The main limitation is the exclusion of patients with poor general health not able to attend the planned exercise intervention. Another limitation is the lack of the gQOL scores before diagnosis and treatment. However, it would be almost impossible to overcome this limitation in any study design. Moreover, in partial correlation networks definite conclusions about the nature and direction of interactions cannot be drawn. The observed connections can represent either direct causal relations or reciprocal causation, can be the result of latent common causes not accounted for in the study or arise from semantic overlap between scales. Future research may endeavour to reproduce these results and can be extended to examine the impact of sociodemographic, lifestyle, clinical and treatment factors. Additionally, the effect of various personality traits (e.g. optimism, conscientiousness, meaningfulness, etc.) on the network structure should be investigated.

## Conclusions

The graphical LASSO networks give us information that may be of help to plan interventions during the early rehabilitation period of breast cancer patients during the treatments and follow-up. In targeting interventions aimed at improving health-related QOL it is important to consider both psychological support and interventions that improve physical function, such as exercise.

## Supplementary Information

Supplementary Information.

## Abbreviations

BDI: Finnish modified version of Beck's 13-item depression scale
BREX: Breast cancer and exercise trial
CS-coefficient: Correlation stability coefficient
gQoL: EORTC C30 global health status / quality of life scale
QOL: Health related quality of life (in general)
EORTC QLQ-C30: European Organization for Research and Treatment of Cancer Quality of Life Questionnaire: Core questionnaire
EORTC QLQ-BR23: European Organization for Research and Treatment of Cancer Quality of Life Questionnaire: BReast cancer module
