# Supplementary material for: A graphical LASSO analysis of global quality of life, sub scales of the EORTC QLQ-C30 instrument and depression in early breast cancer
Source: Sci Rep. 2022 Feb 8;12:2112. doi: 10.1038/s41598-022-06138-2 (PMC8826362; doi:10.1038/s41598-022-06138-2)
Supplement: Supplementary file 1 — Supplementary Information. [file 41598_2022_6138_MOESM1_ESM.docx]

**SUPPLEMENTARY MATERIAL**

A graphical LASSO analysis of global quality of life, sub scales of the EORTC QLQ-C30 instrument and depression in early breast cancer

**Paula Poikonen-Saksela^1#*^, Eleni Kolokotroni^2#^, Leena Vehmanen^1^, Johanna Mattson^1^, Georgios Stamatakos^2^,Riikka Huovinen^3^, Pirkko-Liisa Kellokumpu-Lehtinen^4^ Carl Blomqvist^1^, Tiina Saarto^1^**

^1^Comprehensive Cancer Center, Helsinki University Hospital and University of Helsinki, Helsinki, Finland

^2^In Silico Oncology and In Silico Medicine Group, Institute of Communication and Computer Systems, School of Electrical and Computer Engineering, National Technical University of Athens, Athens, Greece

^3^Department of Oncology, Turku University Hospital and Faculty of Medicine, University of Turku, Turku, Finland.

^4^Faculty of Medicine and Health Technology, Tampere University and Research, Development and Innovation Center, Tampere University Hospital, Tampere, Finland

^#^contributed equally

*corresponding author

**SUPPLEMENTARY MATERIAL SECTION SA**

***Clinical characteristics at baseline***

**Supplement Table 1**. Clinical characteristics at baseline. Total number of patients n=487

| **Variable** | **Mean (range)** |
| --- | --- |
| Age | 52.54 (35-68) |
| **Variable** | **n (%)** |
| pT – tumour size |  |
| T1 | 265 (54.4%) |
| T2 | 184 (37.8%) |
| T3 | 30 (6.2%) |
| T4 | 6 (1.2%) |
| Tis | 1 (0.2%) |
| Tx | 1 (0.2%) |
| pN –number of axillary lymph nodes with cancer |  |
| N0 | 163 (33.5%) |
| N1 | 185 (38.0%) |
| N2 | 54 (11.1%) |
| N1mi | 46 (9.4%) |
| N3 | 22 (4.5%) |
| N0i+ | 17 (3.5%) |
| Estrogen receptor |  |
| Positive | 401 (82.3%) |
| Negative | 86 (17.7%) |
| Progesterone receptor |  |
| Positive | 325 (66.7%) |
| Negative | 162 (33.3%) |
| HER2 (human epidermal growth factor receptor 2) |  |
| Positive | 96 (19.7%) |
| Negative | 391 (80.3%) |
| Breast surgery |  |
| Breast-conserving | 235 (48.3%) |
| Mastectomy | 252 (51.7%) |
| Adjuvant chemotherapy |  |
| Yes | 445 (91.4%) |
| No | 42 (8.6%) |
| Endocrine therapy |  |
| Yes | 405 (83.2%) |
| No | 82 (16.8%) |
| Radiotherapy |  |
| Yes | 384 (78.9%) |
| No | 103 (21.1%) |
| Menopause status before cancer diagnosis |  |
| Postmenopausal | 262 (53.8%) |
| Premenopausal | 225 (46.2%) |
| Trastuzumab treatment |  |
| Yes | 78 (16.0%) |
| No | 409 (84.0%) |

Abbreviations:

T1= 1-10mm, T2= 11-20mm, T3= 21-50mm, T4>51mm, N0=0, N1=1-3, N2=4-9, N1Mi=micro metastases in the axillary lymph nodes, N3>10, or sub clavicular lymph nodes with cancer, N0i+= isolated tumour cells in the axillary lymph nodes

**SUPPLEMENTARY MATERIAL SECTION SB**

***Network stability and accuracy***

Supplement Figures 1B, D shows the average correlation between the node centralities of the original glasso networks and the ones of the bootstrapped subsets as a function of the size of the subsets. Supplement Table 2 contains the CS coefficients for edge weights and node centralities for the glasso networks. Results indicate that the order of node strength and edge weight are stable under subsetting cases for both time points (*CS*(cor=0.7) > 0.5). The stability of closeness is moderate for both time points (*CS*(cor=0.7) > 0.25). On the other hand, CS-coefficients for betweenness fail to reach the minimum threshold of 0.25, indicating unreliable estimates for both time points. Thus, results are interpretable for edge weights, node strength and closeness at baseline and month 12. Supplement Figure 1A, C reveals small to medium 95% CIs around edge weights. The results of the significant difference test show that many edges (Supplement Figure 2) and node strengths (Supplement Figure 3) significantly differ from one-another. Overall, the results indicate that the networks are fairly stable and accurately estimated and support the findings of the analysis of the network structure. Network at baseline is more stable than month 12.

**Supplement Table 2:** Correlation stability (CS) coefficients for node centralities and edge weights for EBIC glasso networks at baseline and month 12

|  | **betweenness** | **closeness** | **edge weight** | **strength** |
| --- | --- | --- | --- | --- |
| M0 | 0.13 | 0.36 | 0.75 | 0.59 |
| M12 | 0.00 | 0.28 | 0.67 | 0.67 |


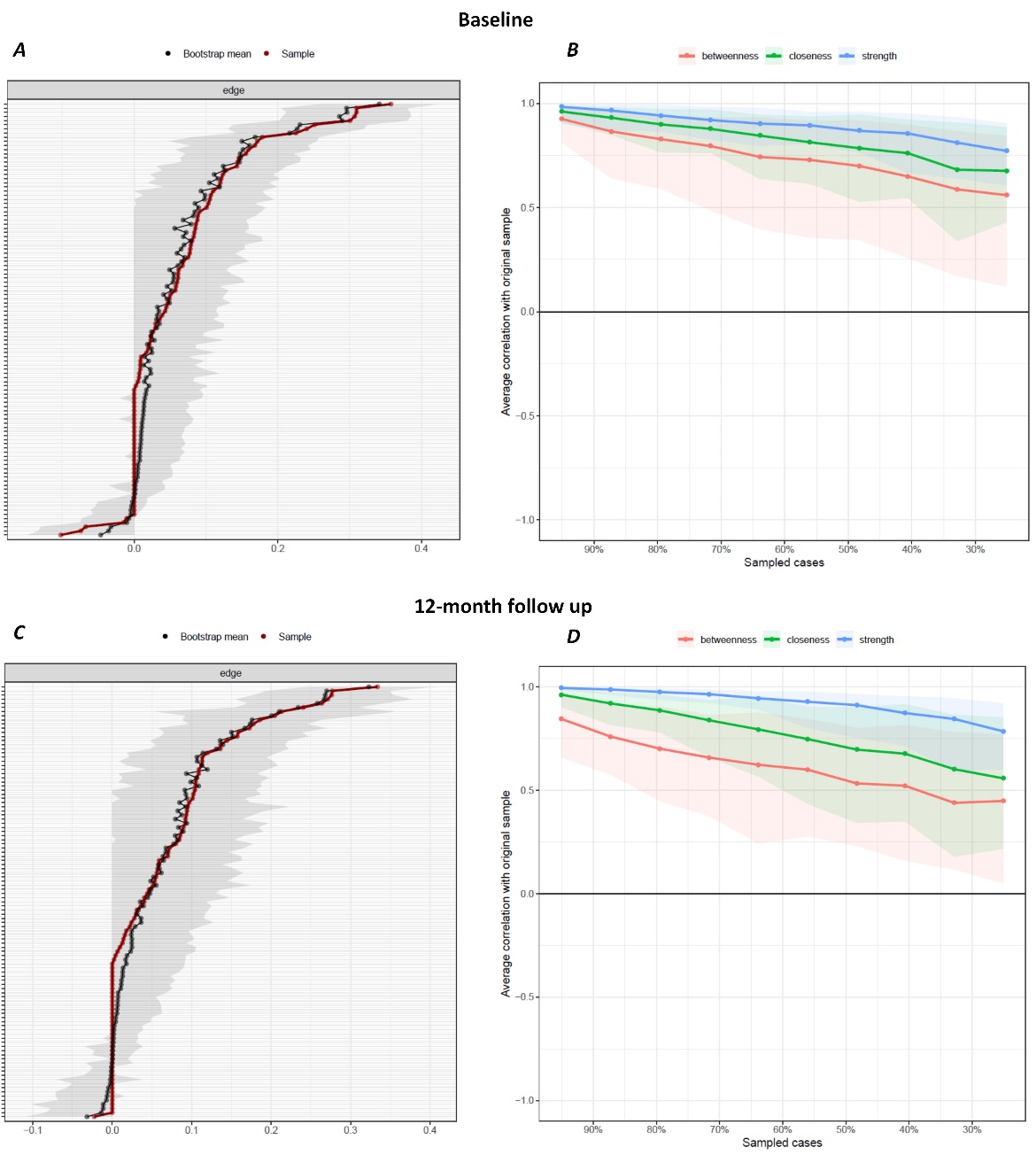


**Supplement Figure 1. Panels A and C: Accuracy of the edge-weights at baseline and 12-month follow up.**

They depict bootstrapped confidence intervals constructed around the edge-weights of the glasso networks, at baseline and 12-month follow up. The red line indicates the sample value of the edge-weight, the black line the bootstrap mean and the grey area the 95% confidence intervals. Each horizontal line represents one edge of the network, ordered from the edge with the highest edge-weight to the edge with the lowest edge-weight.

**Panels B and D: Stability of centrality indices at baseline and 12-month follow up.** They depict the average correlation between the centrality indices of the original network and the ones calculated from a subsample of patients derived via case-dropping subset bootstrap. Lines indicate the means and areas indicate the range from the 2.5th quantile to the 97.5th quantile.


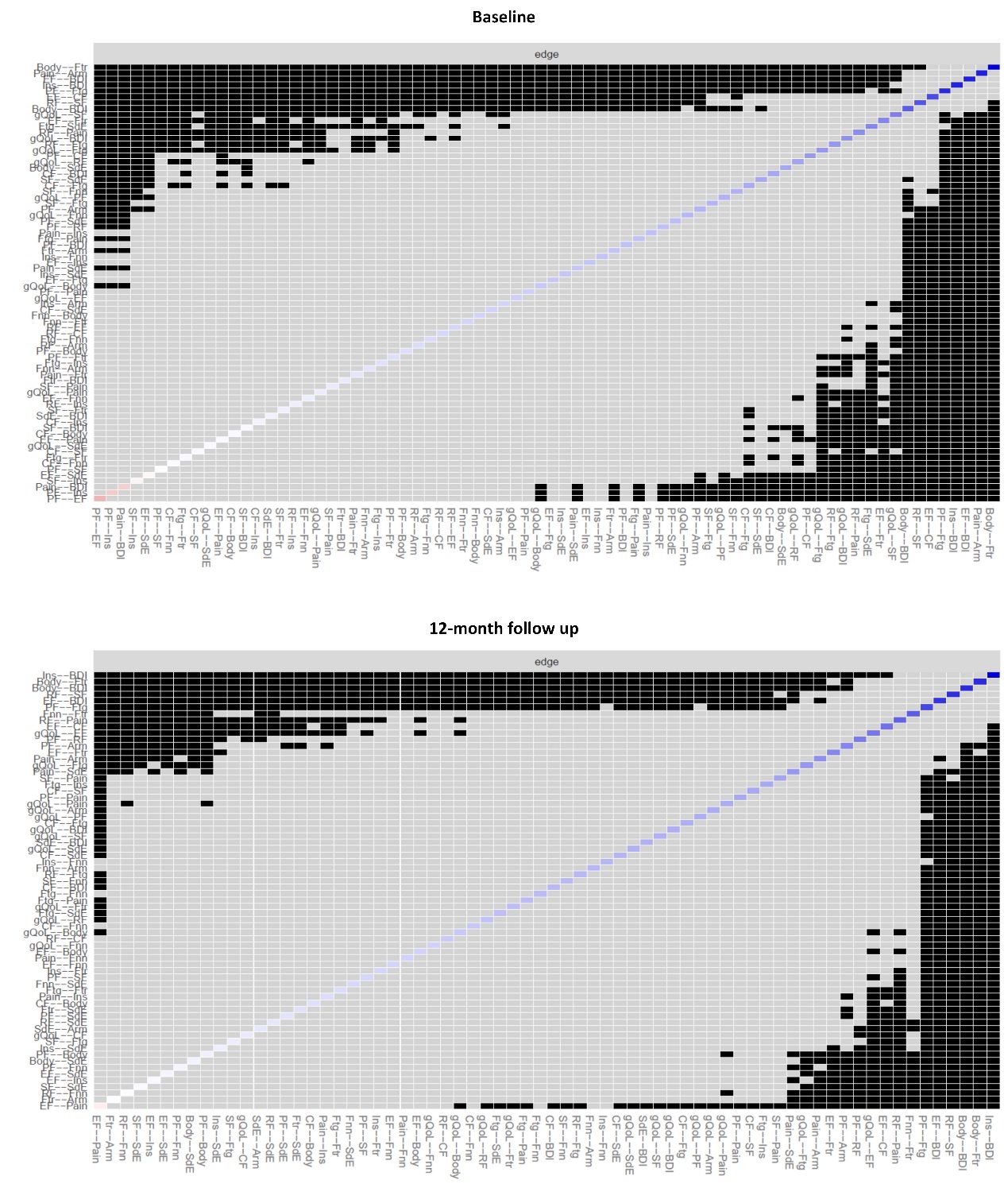


**Supplement Figure 2. Bootstrapped difference tests (α = 0*.*05) between non-zero edge-weights in the glasso networks at baseline and at 12-month follow up.**

Black boxes represent pair of edges that have a significant difference in weight and gray boxes indicate pair of edges that do not have a significant difference in weight. Blue boxes correspond to edges having a positive weight (positive partial correlation between connected nodes) and red boxes correspond to edges having a negative weight (negative partial correlation between connected nodes).

The BDI depression score and the C30/BR23 symptom scores have been reversed to follow the C30/BR23 functioning scales interpretation, i.e. higher score indicates a lower level of symptoms and a better state of the patient. Abbreviations: **gQoL**: C30 Global Quality of Life, **PF**: C30 Physical functioning, **RF**: C30 Role functioning, **SF**: C30 Social functioning, **CF**: C30 Cognitive functioning, **EF**: C30 Emotional functioning, **BDI**: BDI Depression score, **Ftg**: C30 Fatigue, **Fnn**: C30 Financial Difficulties, **Ftr**: BR23 Future perspective, **Body**: BR23 Body image, **Pain**: C30 Pain, **SdE**: BR23 Systemic therapy side effects, **Ins**: C30 Insomnia, **Arm**: BR23 Arm symptoms.


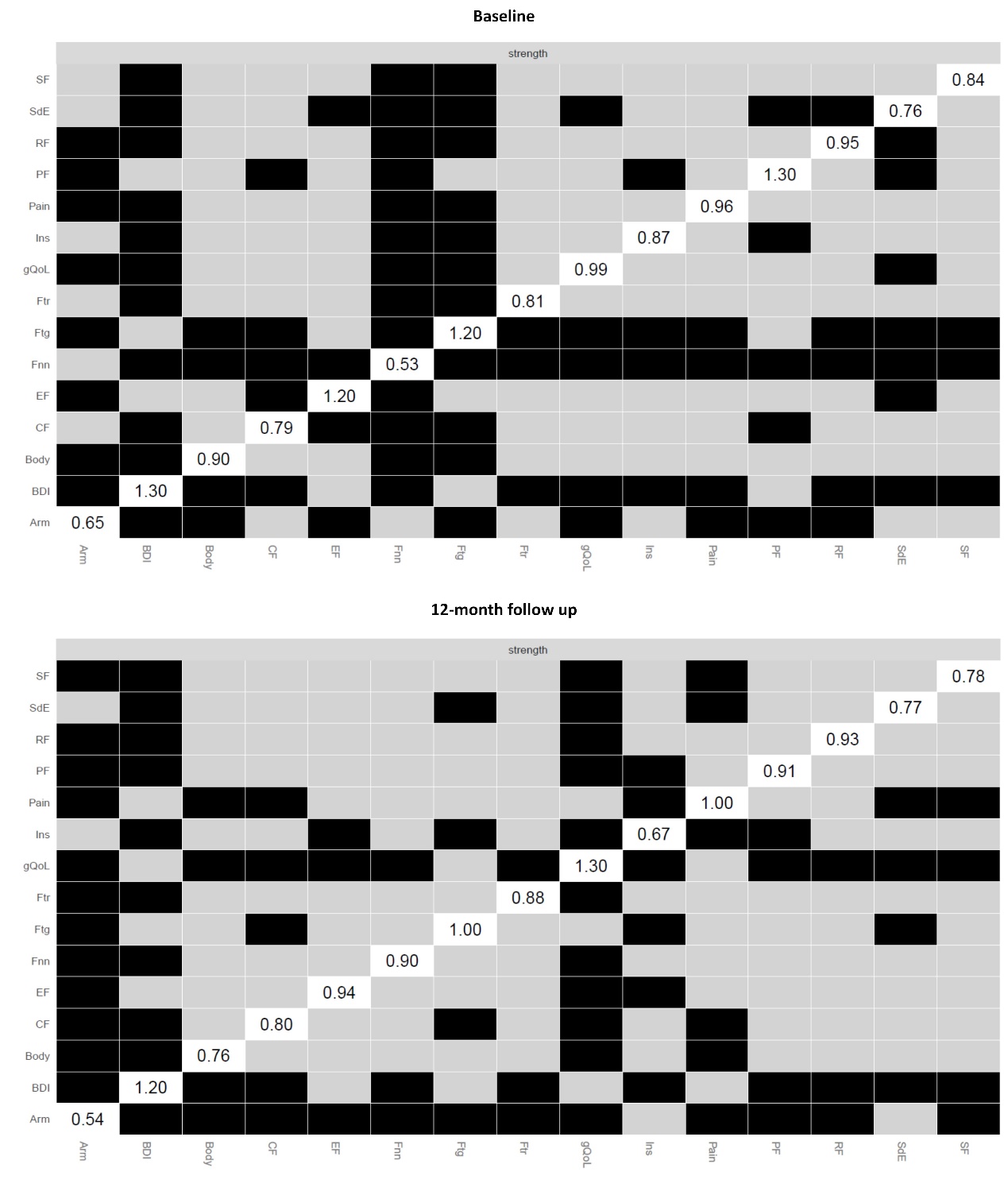


**Supplement Figure 3. Bootstrapped difference tests (α = 0*.*05) between node strength in the glasso networks at baseline and at 12-month follow up.**

Black boxes represent pair of nodes that significantly differ from one another in terms of in strength and grey boxes indicate pair of nodes that do not significantly differ from one another. White boxes show the value of the node strength.

The BDI depression score and the C30/BR23 symptom scores have been reversed to follow the C30/BR23 functioning scales interpretation, i.e. higher score indicates a lower level of symptoms and a better state of the patient. Abbreviations: **gQoL**: C30 Global Quality of Life, **PF**: C30 Physical functioning, **RF**: C30 Role functioning, **SF**: C30 Social functioning, **CF**: C30 Cognitive functioning, **EF**: C30 Emotional functioning, **BDI**: BDI Depression score, **Ftg**: C30 Fatigue, **Fnn**: C30 Financial Difficulties, **Ftr**: BR23 Future perspective, **Body**: BR23 Body image, **Pain**: C30 Pain, **SdE**: BR23 Systemic therapy side effects, **Ins**: C30 Insomnia, **Arm**: BR23 Arm symptoms.

**SUPPLEMENTARY MATERIAL SECTION SC**

***Correlation coefficients***

**Supplement Table 3: Correlation coefficients of EBIC glasso networks at baseline and 12 months follow up.** The BDI depression score and the C30/BR23 symptom scores have been reversed to follow the C30/BR23 functioning scales interpretation, i.e. higher score indicates a lower level of symptoms and a better state of the patient. Abbreviations: **gQoL**: C30 Global Quality of Life, **PF**: C30 Physical functioning, **RF**: C30 Role functioning, **SF**: C30 Social functioning, **CF**: C30 Cognitive functioning, **EF**: C30 Emotional functioning, **BDI**: BDI Depression score, **Fatigue**: C30 Fatigue, **Financial**: C30 Financial Difficulties, **Future**: BR23 Future perspective, **Body**: BR23 Body image, **Pain**: C30 Pain, **SideEffects**: BR23 Systemic therapy side effects, **Insomnia**: C30 Insomnia, **Arm**: BR23 Arm symptoms.

*Edges with absolute weight below 0.05 (in italics) are not displayed in Fig1.

**SUPPLEMENTARY MATERIAL SECTION SD**

***Network Comparison Test***

**Supplement Table 4.** p-values per edge from the permutation test concerning differences in edges weights between the networks at baseline and month 12. Edges appear in increasing order of p-value. Pair of nodes that are not connected by edge in both networks do not appear in the table.

| **Node 1** | **Node 2** | **p-value** | **Node 1** | **Node 2** | **p-value** | **Node 1** | **Node 2** | **p-value** |
| --- | --- | --- | --- | --- | --- | --- | --- | --- |
| Pain | Arm | 0.006 | EF | Body | 0.155 | Fnn | SdE | 0.451 |
| EF | Pain | 0.009 | SdE | BDI | 0.155 | Fnn | Arm | 0.457 |
| QoL | Arm | 0.01 | Pain | Ftr | 0.158 | Pain | Ins | 0.468 |
| EF | SdE | 0.012 | Ftr | Arm | 0.166 | PF | SF | 0.481 |
| Ins | Arm | 0.013 | Ftr | BDI | 0.199 | Ftg | Ftr | 0.481 |
| PF | CF | 0.014 | CF | Ins | 0.204 | SdE | Arm | 0.481 |
| PF | BDI | 0.017 | RF | Ins | 0.23 | CF | Body | 0.488 |
| RF | EF | 0.018 | Ftg | Ins | 0.231 | Ftr | SdE | 0.491 |
| Pain | BDI | 0.023 | PF | RF | 0.255 | PF | Pain | 0.493 |
| EF | Ftg | 0.024 | QoL | SF | 0.258 | RF | Pain | 0.533 |
| PF | Ins | 0.024 | Body | BDI | 0.258 | CF | SdE | 0.544 |
| PF | EF | 0.037 | Fnn | Body | 0.264 | PF | Body | 0.545 |
| Fnn | Ftr | 0.055 | SF | Ftg | 0.279 | Ftg | Fnn | 0.552 |
| QoL | EF | 0.058 | PF | Arm | 0.286 | EF | Fnn | 0.588 |
| Body | SdE | 0.066 | EF | CF | 0.294 | QoL | Fnn | 0.594 |
| CF | SF | 0.075 | PF | Ftg | 0.303 | CF | BDI | 0.618 |
| SF | BDI | 0.092 | EF | Ins | 0.304 | RF | SF | 0.68 |
| QoL | SdE | 0.099 | QoL | BDI | 0.306 | Ins | BDI | 0.683 |
| PF | Ftr | 0.101 | RF | Arm | 0.335 | SF | Fnn | 0.782 |
| SF | Pain | 0.109 | Pain | SdE | 0.347 | EF | Ftr | 0.808 |
| QoL | CF | 0.112 | Ins | SdE | 0.371 | CF | Ftg | 0.835 |
| SF | SdE | 0.114 | PF | SdE | 0.374 | RF | Fnn | 0.841 |
| Body | Ftr | 0.122 | PF | Fnn | 0.377 | RF | CF | 0.86 |
| QoL | Pain | 0.123 | RF | Ftg | 0.386 | Ins | Fnn | 0.886 |
| QoL | Ftr | 0.141 | RF | SdE | 0.407 | QoL | Ftg | 0.89 |
| Pain | Fnn | 0.147 | EF | BDI | 0.415 | QoL | Body | 0.933 |
| CF | Fnn | 0.149 | QoL | RF | 0.439 | Ftg | Pain | 0.984 |
| Ftg | SdE | 0.149 | SF | Ftr | 0.443 | QoL | PF | 0.988 |
| SF | Ins | 0.153 | Ins | Ftr | 0.451 |  |  |  |

**Supplement Table 5.** p-values per node from the permutation test concerning differences in strength centralities between the networks at baseline and month 12.

| **Node** | **p-value** |
| --- | --- |
| QoL | 0.009 |
| PF | 0.022 |
| RF | 0.848 |
| EF | 0.075 |
| CF | 0.893 |
| SF | 0.614 |
| Ftg | 0.018 |
| Pain | 0.523 |
| Ins | 0.072 |
| Fnn | 0.001 |
| Body | 0.154 |
| Ftr | 0.490 |
| SdE | 0.929 |
| Arm | 0.170 |
| BDI | 0.138 |
